# Pigment Biosynthesis and Molecular Genetics of Fruit Color in Pepper

**DOI:** 10.3390/plants12112156

**Published:** 2023-05-30

**Authors:** Linlin Wang, Yangmin Zhong, Jia Liu, Ruifang Ma, Yeminzi Miao, Wenqi Chen, Jiaqiu Zheng, Xin Pang, Hongjian Wan

**Affiliations:** 1 State Key Laboratory for Managing Biotic and Chemical Threats to the Quality and Safety of Agro-Products, Institute of Vegetables, China-Australia Research Centre for Crop Improvement, Zhejiang Academy of Agricultural Sciences, Hangzhou 310021, China; wanglinlin4585@163.com (L.W.); liu-l-jia@163.com (J.L.); chenwenqiawan@163.com (W.C.); 2 Institute of Crops, Lishui Academy of Agricultural and Forestry Sciences, Lishui 323000, China; zhongyangmin2021@126.com (Y.Z.); mareifang@126.com (R.M.); mz13506828816@163.com (Y.M.); 3 Jiangsu Coastal Area Institute of Agricultural Sciences, Yancheng 224002, China; nky8236@163.com; 4 Suzhou Polytechnic Institute of Agriculture, Suzhou 215008, China

**Keywords:** pepper, fruit color, biosynthesis, molecular genetics, research progress

## Abstract

Pepper, as a vegetable crop with a wide cultivation area worldwide, besides being a significant condiment and food, also has a momentous use for chemistry, medicine, and other industries. Pepper fruits are rich in various pigments, such as chlorophyll, carotenoids, anthocyanins, and capsanthin, which have important healthcare and economic value. Since various pigments are continuously metabolized during the development of pepper fruits, peppers exhibit an abundant fruit-colored phenotype in both the mature and immature periods. In recent years, great progress has been made in the study of pepper fruit color development, but the developmental mechanisms are still unclear systematically dissected in terms of pigment, biosynthesis, and regulatory genes. The article outlines the biosynthetic pathways of three important pigments: chlorophyll, anthocyanin, and carotenoid in pepper and the various enzymes involved in these pathways. The genetics and molecular regulation mechanisms of different fruit colors in immature and mature peppers were also systematically described. The objective of this review is to provide insights into the molecular mechanisms of pigments biosynthesis in pepper. This information will provide theoretical basis for the breeding of high-quality colored pepper varieties in the future.

## 1. Introduction

Pepper (*Capsicum annum* L.), a member of the Solanaceae family, originated in tropical Latin America and is one of the extensive cultivated vegetable crops worldwide [1]. According to the Food and Agriculture Organization of the United Nations (FAO), global pepper production in 2020 exceeded 39 million tons (FAO, 2020). Pepper is closely bound up with human life. It is not only an essential fresh vegetable and condiment in people’s meals but also an important industrial raw material. The capsanthin and capsorubin derived from pepper have significant uses in the food, chemical industry, and agriculture [2]. Meanwhile, colorful ornamental peppers are gradually emerging in the selection of landscape plants [3]. Therefore, peppers have significant economic and application value.

Pepper fruit quality is determined by multiple traits, affecting visual presentation, flavor, chemical composition, and nutritional value. Among these quality traits, fruit color is of primary importance since the pigments that confer color are related to nutrition, health, and flavor. Carotenoids can be used as dietary sources of provitamin A for humans [4]. Lycopene protects against cardiovascular disease and possibly prostate cancer [5]. Deauxogenated carotenoids produced by oxidative cleavage of carotenoid dioxygenase have a series of important biological activities, which can produce unique flavors and aromas [6].

The color of pepper fruit is one of the most intuitive and essential quality traits influencing consumer and breeder preferences during the purchase and cultivation process. Breeders have bred peppers with different peel colors through beneficial genes and mutations to increase the diversity of commodities while meeting various consumer needs [7]. New varieties of peppers with different fruit colors, such as white, purple, yellow, black, and orange, have been bred to date.

Fruit ripening is often accompanied by a marked color shift involving three mechanisms: (1) loss of chlorophyll from chloroplasts, accompanied by the decomposition and recycling of some thylakoid membranes and photosynthetic proteins. (2) Accumulation of colored carotenoids in lipid spheres or other characteristic membrane-bound structures in developing chloroplasts, which are transformed by chloroplasts or develop from other types of plastids. (3) Accumulation of flavonoid or anthocyanin-like pigments in cytosolic vesicles [8,9,10,11]. The different mechanisms are not mutually exclusive, and several mechanisms can act on the same fruit. For example, during ripening in some apples, chloroplasts in pericarp cells lose chlorophyll and accumulate carotenoids and sometimes anthocyanins, although some varieties remain green [12,13]. Tomatoes usually lose chlorophyll and accumulate carotenoids, but “*green flash*” mutant fruits retain some thylakoids and chlorophylls while accumulating carotenoids, therefore having dark brown fruits [14,15]. Strawberries, black grapes, and Chinese bayberry are fruits that lose chlorophyll and usually accumulate only anthocyanins, so the fruits appear a red or black color [16,17]. Lutein is the main carotenoid that existed in most citrus fruits which gives them their red hue. Red grapefruit and red-fleshed navel orange are typical mutants that appear red because of the accumulation of lycopene and β-carotene [18]. Capsaicin, a carotenoid unique to pepper fruit, was detected in red and pink ripe fruit, while the pigment was not detectable in yellow fruit or young green fruit [19].

In recent years, the genetics of the pepper genome have also advanced, especially with the completion of sequencing and assembly of the pepper genome in 2014, which took a big step forward. Qin et al. [20] completed sequencing the whole genome of C. annuum ‘Zunla 1’ and the wild species ‘Chiltepin’. The sequencing depths were 146.43× and 96.37×, respectively. Then the two material genomes were assembled, and the genome sizes were 3.35 Gb and 3.48 Gb after assembly. At the same time, researchers from Korea conducted 18.86× depth sequencing for CM334, and finished resequencing de novo sequencing for another two cultivated species and one wild species, respectively [21]. The evolution of pepper resources and the absence of spiciness were then studied by comparing the genome sequences of tomatoes and other Solanaceae crops. There has been a rapid increase in research on the development of yield, resistance, and other traits in pepper [22,23,24,25,26]. With the continuous improvement of the pepper genome sequence and the development of genetic maps, research on pepper fruit color will make considerable headway. This has provided new opportunities to analyze the mechanism of fruit color formation in pepper.

Pepper fruit color depends on the relative content of chlorophyll, carotenoid, anthocyanin, and other pigment substances in the pepper fruit. Among them, chlorophyll, anthocyanin, and carotenoid in pepper fruit have specific antioxidant effects, reducing the risk of cancer and cardiovascular diseases [27]. Furthermore, capsaicin, an essential antioxidant substance for scavenging free radicals, is now widely used in food processing, catering, pharmaceutical, and feed industries [28]. The fruit color of pepper is varied and has become a model plant for studying fruit color inheritance through its colorful fruits.

This paper reviewed the research progress of fruit color in pepper. We summarized recent studies on the biosynthesis of pigments in pepper fruits. The genetics and molecular regulation mechanisms of different fruit colors in immature and mature peppers were also systematically described. This study aims to provide valuable insights into the molecular mechanisms underlying pigments biosynthesis in pepper.

## 2. Biosynthetic Pathways of Pigments in Pepper Fruits

### 2.1. Classification of Pigments in Pepper Fruits

**Figure 1:**
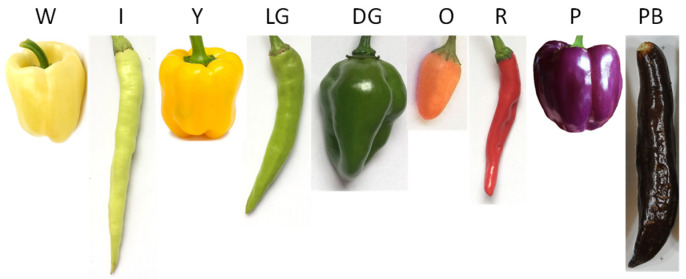
Representative fruit color of pepper. W, White; I, Ivory; Y, Yellow; LG, Light Green; DG, Dark Green; O, Orange; R, Red; P, Purple; PB, Purple Black.

The accumulation and proportion of various pigments in the fruits, such as chlorophylls, anthocyanins, and carotenoids, determine the color of pepper fruits. These pigments impact the color of pepper fruit while also having the effects of scavenging free radicals and improving immunity and antioxidants in the human body. Peppers are of great diversity and rich color (Figure 1). The immature fruit colors include green, red, white, purple, and black. The analysis using high liquid chromatography showed that the shade of green is related to chlorophyll and is also influenced by small amounts of carotenoids such as lutein, β-carotene, and violaxanthin [29]. The white fruits are deficient in chlorophyll and carotenoids [30]. The primary pigment components in purple and black fruits are anthocyanin and chlorophyll, and the pigment content in the black fruits was significantly higher than that in the purple fruits [31]. Red and yellow peppers are exceptionally high in carotenoids [32].

In addition to the varieties and contents of pigments, the colors of the pepper fruits will vary during development due to the continual metabolism of individual peel pigments [33]. Pepper fruits have a rich diversity of fruit colors that alter from pre-mature to mature. One of the most common types changes from pre-mature green to mature red. Some green fruits shift to yellow, olive green, or brown. The ivory color shifted to white, purple to red, orange to red, and the black color to red or orange [34]. It happened due to the tendency of mature fruit colors to lose pigments. Moreover, the fruits change from physiological maturity to overmature and acquire color as they switch from mature green to standard mature fruit color [35].

The mature pepper fruits exhibit various fruit colors, including red, yellow, orange, olive green, and brown colors [36]. Carotenoids play a significant role in the red-ripening fruit color of pepper, as chloroplasts in the pepper fruits differentiate and develop into chromoplasts, during which the gradual degradation of chlorophyll in fruits and the synthesis and accumulation of carotenoids in chromoplasts coincide [37]. In short, the phenotype of mature pepper fruit color is determined by the types and relative contents of carotenoids accumulated in chromoplasts. With the exploration of the color resources from different pepper fruits, it has been found that chlorophyll does not degrade in peppers with brown and green fruits during the ripening process [38]. Mature peppers with brown fruits are affected by a combination of chlorophyll and red carotenoids [39].

### 2.2. Pathway of Pigment Biosynthesis in Pepper Fruits

#### 2.2.1. Biosynthetic Pathway of Chlorophyll

**Figure 2:**
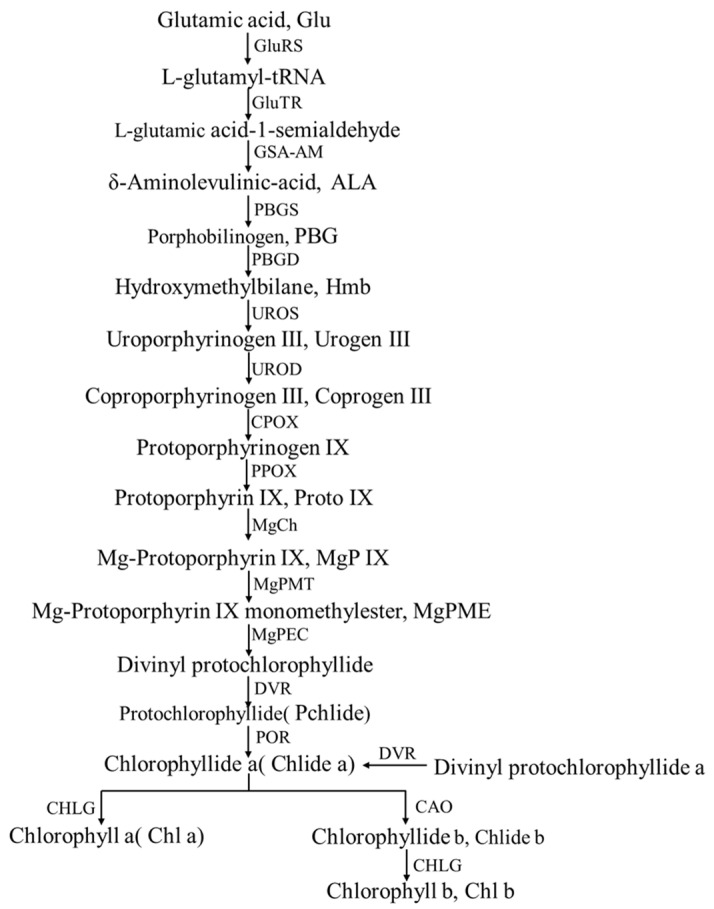
Biosynthetic pathway of chlorophyll. Key enzymes in anthocyanin biosynthetic pathway. GluTR: Glutamyl-tRNA reductase; GSA-AM: Glutamate-1-semialdehyde 2, 1-aminomutase; PBGS: Porphobilinogen synthase; ALAD: 5-Aminolevulinate dehydratase; PBGD: Porphobilinogen deaminase (Hydroxymethylbilane synthase); UROS: Uroporphyrinogen III synthase (Uroporphyrinogen III co-synthase); UROD: Uroporphyrinogen III decarboxylase; CPOX: Coproporphyrinogen III oxidase; PPOX: Protoporphyrinogen oxidase; MgCh: Magnesium chelatase H subunit, Magnesium chelatase I subunit, Magnesium chelatase D subunit; MgPMT: Magnesium proto IX methyltransferase; MgPEC: Mg-protoporphyrin monomethylester; DVR: 3,8-Divinyl protochlorophyllide a 8-vinyl reductase; POR: Protochlorophyllide oxidoreductase; CHLG: Chlorophyll synthase; CAO: Chlorophyllide a oxygenase.

Chlorophyll (Chl) is composed of the magnesium-containing porphyrin ring and phytol, mainly including chlorophyll a and chlorophyll b. The chlorophyll in pepper contains a vinyl group at the C-3 position and an ethyl group at the C-8 position in the tetrapyrrole macrocycle, which belongs to monovinyl chlorophyll (MV-Chl) [24]. Chlorophyll synthesis in peppers can be divided into three major stages (Figure 2).

The first stage is the synthesis from L-glutamyl to δ-aminolevulinic acid (ALA), which begins with the catalytic reaction of glutamic acid (Glu) and glutamyl-tRNA synthetase (GluRS). L-glutamyl-tRNA is the main product of this catalytic reaction. The above reaction is generally considered the starting point of Chl biosynthesis [40]. L-glutamyl-tRNA was then reduced to L-glutamic acid 1-semialdehyde by glutamyl-tRNA reductase (GluTR), releasing complete tRNA simultaneously; glutamate-1-semialdehyde is catalyzed by L-glutamate-1-semialdehyde2,1-aminomutase (GSA-AM) to form ALA, which is encoded by the gene GSA [40,41]. The enzymes involved in the reaction at this stage do not react individually. The individual enzymes involved in this reaction phase do not react independently but form a complex with each other, intended to inhibit competition for glutamate-tRNA from other substances’ synthesis mechanisms [42].

The second stage is the synthesis of protoporphyrin IX (Proto IX) from ALA. ALA is catalyzed and condensed by porphobilinogen synthase (PBGS) to form porphobilinogen (PBG). PBG is then catalyzed by porphobilinogen deaminase (PBGD) to produce hydroxymethylbilane (Hmb). Coproporphyrinogen III (Coprogen III) is obtained from Hmb by the action of uroporphyrinogen III synthase (UROS) and uroporphyrinogen III decarboxylase (UROD). Finally, coprogen III undergoes a two-step oxidation reaction by coproporphyrinogen III oxidase (CPOX) and protoporphyrinogen oxidase (PPOX) to form Proto IX [43,44]. The pre-two stages of Chl biosynthesis share the same synthetic pathway as tetrapyrrole substances in plants, such as photosensitive pigments. After the formation of Proto IX, it is the pathway of chlorophyll synthesis with other substances, which also means that chlorophyll synthesis enters the third stage [45].

The third stage is the synthesis of Chl, and the magnesium chelatase reaction at this stage is a sign of Proto IX entering the chlorophyll synthesis pathway. Accordingly, magnesium chelatase (MgCh) has become one of the critical enzymes in whole chlorophyll biosynthesis [46]. Proto IX synthesizes chlorophyll a by the action of Mg Ch, Mg-protoporphyrin IX methyltransferase (Mg PMT), Mg-protoporphyrinogen IX monomethylester cyclase (Mg PEC), 3,8-Divinyl protochlorophyllide an 8-vinyl reductase (DVR), protochlorophyllide oxidoreductase (POR), chlorophyll synthase (Childe), chlorophyll synthase (CHLG) and other enzymes. Finally, the methyl of the C7 side chain of chlorophyll a is oxidized to form a formyl by chlorophyllide oxygenase (CAO), which completes the synthesis of chlorophyll b [47,48].

The steps of the chlorophyll biosynthesis process are tedious and complex, among which the synthesis of ALA and the insertion of Mg ions are the two main processes controlling chlorophyll biosynthesis. Meanwhile, the whole process of chlorophyll biosynthesis is not only regulated by intrinsic genes but also by external environmental conditions [49,50]. However, the regulatory mechanism of these factors is still unclear, and continued intensive research is required.

#### 2.2.2. Biosynthetic Pathway of Anthocyanin

Anthocyanin is a crucial pigment that mainly exists in the tissue vesicles of flowers, fruits, leaves, and stems of peppers. Furthermore, this anthocyanin gives pepper fruits their purple or nearly black colors [51]. Anthocyanin is a product of the secondary metabolism of plant polyphenolic flavonoids with a typical skeletal structure of flavonoids. Anthocyanins have also been shown to have anticancer, antiallergic, anti-inflammatory, antiviral, and antioxidant properties, implying that anthocyanins have health benefits in addition to being essential pigments that determine plant color [52].

**Figure 3:**
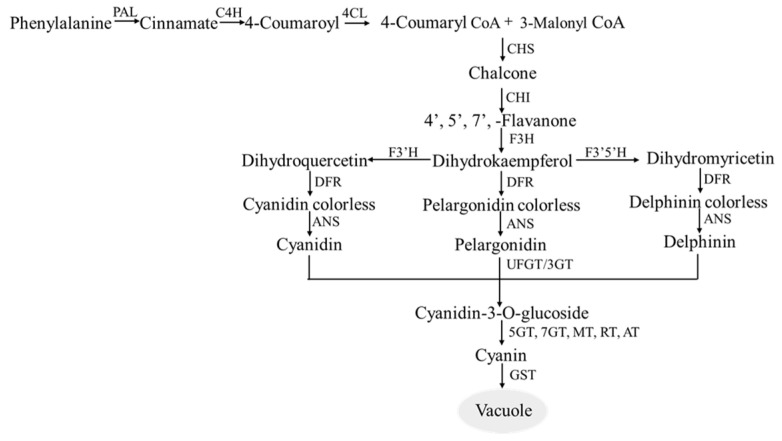
Biosynthetic pathway of anthocyanin. Key enzymes in anthocyanin biosynthetic pathway. PAL: Phenylalanine ammonia-lyase; C4H: Cinnamate 4-hydroxy-lase; 4CL: 4-counmaric CoA ligase; CHS: Chalcone synthase; CHI: Chalcone isomeras; F3H: Flavanone-3-hydroxylase; F3′H: Flavanone-3′-hydroxylase; F3′5′H: Flavanone-3′,5′-hydroxylase; DFR: Dihydroflavonol-4-reductase; ANS: Anthocyanidin synthase; UFGT: Flavonoid 3-O-glucosyltransferase; 5GT: Flavonoid 5-O-glucosyltrasferase; 7GT: Flavonoid 7-O-glucosyltransferase; MT: Methyltransferase; RT: Rhamnosyltransferase; AT: Acyltransferase; GST: Glutathione S-transferase.

Although the anthocyanin biosynthesis pathway and the main components are not the same in different pepper materials, the expression of genes related to the synthesis of anthocyanins has been changing during the development of pepper fruits [53]. It can be determined from previous studies that anthocyanin biosynthesis is an important branch of the flavonoid metabolic pathway. The anthocyanin biosynthesis pathway in plants can be divided into three stages (Figure 3).

The first stage is the phenylpropanoid metabolic reaction, a necessary secondary metabolic pathway shared by most plants. As a precursor for the biosynthesis of anthocyanins and other flavonoids, phenylalanine utilizes phenylalanine ammonia-lyase (PAL), cinnamate 4-hydroxylase (C4H), and 4-coumarate Co A ligase (4CL) to generate 4-coumaroyl CoA through an initial metabolic reaction. 

The second stage is a key step in the metabolism of flavonoids, where acetyl-CoA carboxylase (ACC) and acetyl CoA ligase (ACL) catalyze acetic acid to give malonyl CoA, which is then combined with the 4-coumaryl CoA formed in the previous first stage. The products are catalyzed by chalcone synthase (CHS) to form chalcone, which is catalyzed and converted by chalcone isomerase (CHI) and flavanone 3-hydroxylase (F3H) to obtain dihydroflavonol. Then the hydroxylate is continued by flavonol 3′ hydroxylase (F3′H) and flavonol 3′,5′ hydroxylase (F3′5′H) at different sites to form dihydroquercetin (DHQ) with dihydromyricetin (DHM). 

The third stage is the reaction stage of various plant anthocyanins. Colorless dihydroflavonol, DHM, and DHQ are catalyzed by dihydroflavonol 4-reductase (DFR) to form colorless anthocyanins, which are catalyzed by anthocyanidin synthase (ANS) and leucoanthocyanidin dioxygenase (LDOX) to synthesize different colored anthocyanins. Since anthocyanins are not stable and usually do not exist in the free state, they are bound to glycosyl groups in the form of glycosides. Therefore, anthocyanin synthesized through the above steps is catalyzed by flavonoid 3-O-glucosyltransferase (UFGT) to combine with glycoside to form colored anthocyanin. Anthocyanins are transported by glutathione S-transferase (GST) and stored in vacuoles [54,55,56,57].

Although significant progress has been made in understanding the biosynthetic pathways of anthocyanins, a more comprehensive regulatory network remains to be explored. The accumulation of anthocyanins is dependent on the balance between biosynthesis and degradation; however, the mechanism underlying anthocyanin degradation remains unclear. Furthermore, environmental factors such as light, temperature, and glucose metabolism have an impact on anthocyanin metabolism and their regulatory mechanisms require further investigation.

#### 2.2.3. Biosynthetic Pathway of Carotenoid

Carotenoid is a type of ethylene pentadiene compound associated with the coloration of petals and fruits in many horticultural crops, such as tomatoes, peppers, marigolds, and oranges [58]. There is a wide variety of carotenoids, and the types of carotenoids present in mature pepper fruits are diverse. Carotenoids in ripe pepper fruits are capsaicin, capsanthin, and other flavonoid lutein; zeaxanthin, violaxanthin, and other lutein [59]. There is no capsaicin or capsorubin in yellow and orange ripe pepper fruits, but rather luteolin substances such as violaxanthin, luteolin, and violet flavin [60]. The carotenoids in olive ripe pepper fruits are mainly yellow carotenoids, while the carotenoids in brown ripe pepper fruits are red carotenoids such as lutein and capsaicin [61,62]. 

**Figure 4:**
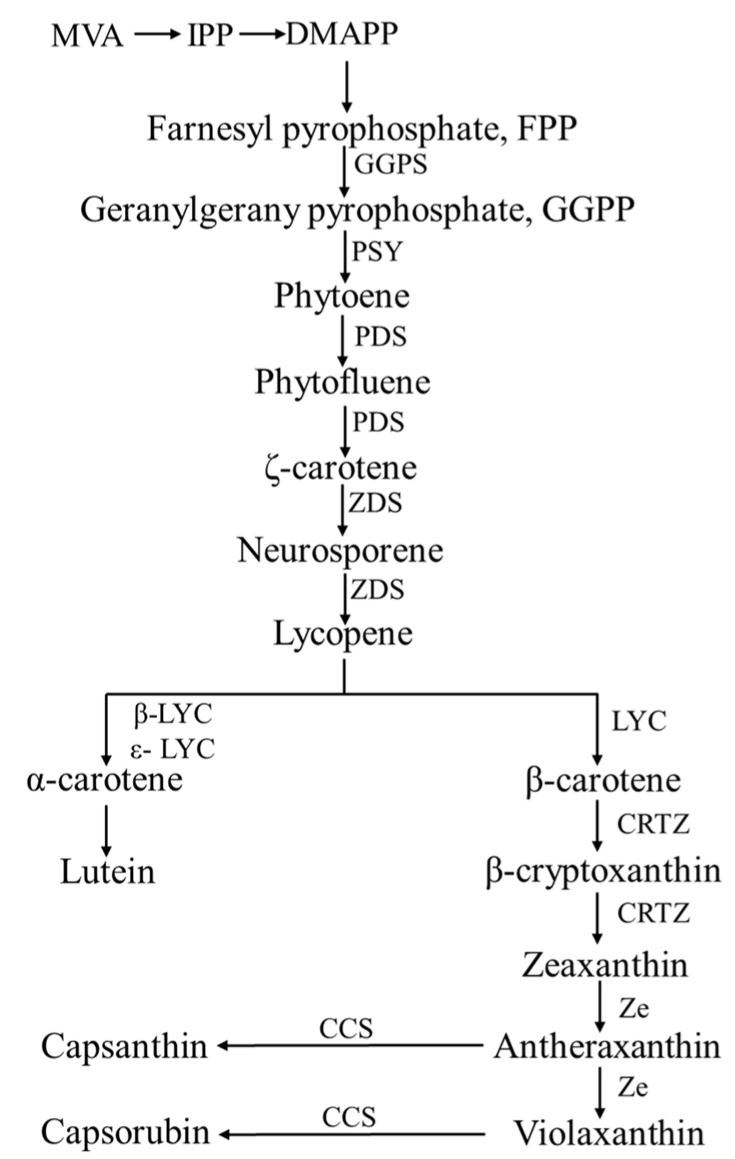
Biosynthetic pathway of carotenoid. Key enzymes in carotenoid biosynthetic pathway. GGPS: Geranylgeranyl pyrophosphosphate synthase; PSY: Phytoene synthase; PDS: Phytoene desaturase; ZDS: ζ-carotene desaturase; LYC: Lycopene cyclase; β-LYC: Lycopene β-cyclase; ε-LYC: Lycopene ε-cyclase; CRTZ: β-carotene hydroxylase; Ze: Zeaxanthin epoxidase; CCS: Capsanthin-capsorubin synthase.

All the precursors for carotenoid synthesis in pepper are produced through isopentenyl diphosphate (IPP), dimethylallyl diphosphate (DMAPP), and (2E, 6E)-farnesyl diphosphate (FPP). The final product of this action is geranylgeranyl diphosphate (GGPP). GGPP is not only a direct precursor of carotenoids but also a common precursor of various plastid-like ethylene glycols, including geranylgeranyl pyrophosphate, chlorophyll, and tocopherol [63] (Figure 4). 

The lycopene cyclization reaction is the following third stage. It is an important branch of the carotenoid synthesis pathway because the enzymes related to this stage can be divided into two classes in plants and catalyze branching reactions in two different directions. At this stage, lycopene is catalyzed by lycopene β-cyclase (LCY-b) and lycopene-cyclase (LCY-e) to obtain α-carotene. In contrast, the other branch is catalyzed only by LCY-b to obtain β-carotene. At this stage, in addition to the transcriptional regulation of *PSY*, the molecular synergy between LCY-b and LCY-e is the regulatory node regulating carotenoid biosynthesis and accumulation.

In the fourth stage, the 3 and 3′ carbon atoms of the two branching products from the previous stage are hydroxylated by specific enzymes to form zeaxanthin and lutein, respectively. It means that α-carotene is converted to lutein by the combination of β-caro-tene 3-hydroxylase (crtZ) and carotenoidε-hydroxylase (LUT1), while β-carotene is hydroxylated to form zeaxanthin. Because of zeaxanthin’s unstable structure, it is converted into antheraxanthin by zeaxanthin epoxidase (ZEP) and β-ring hydroxylase (LUT5), which can ultimately produce violaxanthin. Finally, capsanthin/capsorubin synthase (CCS) converts antheraxanthin to capsanthin, which can be epoxidized by ZEP and catalyzed by CCS to produce capsorubin. Both are the main pigments affecting the color of ripe pepper fruits [63,64,65].

The biosynthesis processes of chlorophylls, anthocyanins, and carotenoids in peppers are complex, and the synthesis pathway involves a variety of enzymes. Meanwhile, their variation-related genes often lead to differences in the components and contents of various pigments accumulated in the fruits of different pepper resources. At the same time, the synthesis and metabolism of various pigments are also activated and inhibited by their endogenous hormones and external environment to varying degrees. The pepper fruits show different color phenotypes in immature and mature periods finally.

### 2.3. Relationship between Fruit Color and Pigment Formation of Pepper

The composition and content of various pigments in pepper fruits undergo extensive changes in morphology, physiology, and metabolism during ripening. In most pepper fruits, chloroplasts gradually differentiate and develop into chromoplasts as they mature, and carotenoids are synthesized and accumulated in chromoplasts. The color of pepper fruits during ripening is dominated by carotenoid substances. There are exceptions, however, where chloroplasts of brown and olive-green pepper fruits do not degrade [37,66].

The differences in the color of pepper fruits are caused by variations in the type, relative content, synthesis and metabolism of pigments during fruit ripening [67]. The varieties of peppers that turn from green to red have higher contents of total carotenoids and capsaicin at maturity. The varieties that turn from white to red have higher contents of capsaicin and lower contents of total carotenoids at maturity. The varieties that turn from green to orange have higher contents of total carotenoids but no synthesis of capsaicin at maturity. The yellow pepper varieties have lower contents of total carotenoids at maturity. The reason for the brown color of pepper fruits is that the chloroplasts are not degraded. Green and red are superimposed to form brown, and green and yellow are superimposed to form olive green. The green color is chlorophyll and the red color is capsaicin [59,68,69,70].

## 3. Research of Immature Fruit Color in Pepper

### 3.1. Research of Green and White Immature Fruit Related Genes in Pepper

The process of chlorophyll biosynthesis is a multi-step enzymatic reaction that requires 16 steps and involves 15 enzymes (Table 1). Immature pepper fruits show significant natural variation in chlorophyll content and color [71]. In recent years, more and more scholars have conducted studies on green and white immature pepper fruits with remarkable results. Using green and white pepper materials as parents, Chen et al. obtained six generations of peppers by crossing, selfing, and backcrossing, finding that the inheritance of pepper pericarp was consistent with nuclear inheritance [72].

The light and dark green coloration of immature peppers are related to the chlorophyll content in their fruits. Brand et al. [71,73] constructed an F_2_ population derived from the cross between a dark green pepper and a light green pepper. Two major QTLs, *pc8.1* (now renamed *pc1*) and *pc10.1*, controlling the chlorophyll content of pepper fruits were identified. Among them, *pc10.1* affects chlorophyll content by controlling the chloroplast compartment size via modulating grana and chloroplast size, whereas *pc8.1* controls both chloroplast area and number. Subsequently, Borovsky et al. revised *QTL8.1* to *QTL1*. Based on covariance analysis with Solyc08g076940 and Solyc08g077280 and BSA-seq localization, *pc1* was located on chromosome 1 [74]. 

Safi et al. found that *GLK2*, a nuclear-encoded transcription factor belonging to the GARP family, promotes chloroplast development by activating genes encoding proteins related to photosynthetic processes (chlorophyll biosynthesis, light harvesting, and electron transport), which in turn leads to the deepening of green fruit color [75]. By using the gene information of the tomato *u* (uniform ripening) mutant *SIGLK2*, it was found that the pepper orthologous gene *CaGLK2* co-segregated with *pc10*. *CaGLK2* was identified as a candidate gene for *pc10.1* based on genetic analysis of the null allele associated with reduced chlorophyll content and gene expression analysis of *CaGLK2* in dark green fruits through known gene function analysis in tomato and *Arabidopsis* [71,73]. Pan et al. found that the positive regulator of chlorophyll synthesis in tomatoes was identified as the *APRR2-Like* gene, and the increase in chlorophyll of immature tomato fruits was attributed to the positive expression of this gene. Researchers cloned the *APRR2-Like* homologous gene in pepper, and a single base substitution (G/A) that forms a termination codon was found in the white pepper fruit [76]. It was indicated that *CaAPRR2* was closely related to the low chlorophyll content and white phenotype of pepper fruit, and the gene was in the same position as *pc8.1* (*pc1*) on the genetic map [76]. Borovsky et al. reported that *LOL1* (*LSD ONE LIKE1*; *CcLOL1*), a homolog of the pepper zinc finger transcription factor, affected the development of Solanaceae fruits in a fruit-specific manner and interacted with various genes related to photosynthesis and redox in immature pepper fruits to affect the chlorophyll content of fruits [64]. The same study also verified the function of this gene by knocking out the tomato homologous gene to obtain light green tomato fruits. In addition, it was shown that the diversity of alleles and a combination of the three known genes, *LOL1*, *GLK2*, and *APRR2*, which affect the colors of immature fruits, were associated with variations in chlorophyll content in peppers [74].

It has been suggested that three pairs of genes regulated the phenotypic differences in green, yellow-green, and white immature pepper fruits, and green had a dominant epistatic effect [77]. Lightbourn (2008) discovered that peppers with green, yellow-green, and ivory-white immature fruit colors were regulated by a series of *sw* genes, with *sw1* controlling ivory-white, *sw2* controlling yellow-green, and green being controlled by the *sw3* locus, where *sw3* was dominant to *sw1*, *sw2* and *sw2* were dominant to *sw1* [31].

### 3.2. Research of Violet and Nearly Black Immature Fruit Related Genes in Pepper

At present, both violet and nearly black peppers are rare germplasm resources. They have also received attention and research from many researchers. The immature pepper fruit phenotypes of violet and nearly black are mainly influenced by anthocyanin and chlorophyll. Furthermore, the content of both anthocyanin and chlorophyll in black fruits is much higher than that in violet fruits, with the black fruits having a 14-fold chlorophyll content [33]. The complex process of anthocyanin biosynthesis in pepper fruits involves a variety of structural and regulatory genes. However, not all genes play key roles in anthocyanin synthesis, such as *CHS* and *CHI*, which exist in the whole growth process of plants with different genotypes but do not play a decisive role in the synthesis process [78]. Through semi-quantitative expression analysis of nine genes involved in the anthocyanin biosynthesis pathway in pepper fruits at different developmental stages, Aza-Gonzalez et al. identified four key genes: *F3′5′H*, *DFR*, *GST*, and *UFGT*, and the genes coordinated with each other to complete the anthocyanin synthesis in pepper fruits [79]. It has been reported that the expression of each gene in the anthocyanin biosynthesis pathway is regulated by transcription factors (TFs), especially the MBW complex consisting of MYB, bHLH, and WD40 [80]. The MBW complex usually interacts with the promoters of structural genes to up-regulate or decrease their expression, resulting in a significant increase or decrease in anthocyanin content. Moreover, optical signal factors also interact with the MBW complex, affecting the stability of the MBW complex and the anthocyanin synthesis process [80].

The violet pepper fruits are controlled by the incompletely dominant gene *A* (*Anthocyanin*). The *A* gene in peppers is allelic to the *fc* gene that controls the purple anther filaments of peppers, and it is also homologous to *An2* (*Anthocyanin2*) of petunia. *A* is located on chromosome 10 of pepper and is involved in regulating anthocyanin biosynthesis. Moreover, the violet fruit is also regulated by the *MoA* gene located on chromosome 11. The presence or absence of violet immature pepper fruits belongs to the quality regulated by the *A* gene, which can also be considered as a mono-dominant gene relative to green pepper immature fruits if the violet depth of the pepper fruit is not considered, and the *MoA* gene controls the shade of purple color [81,82].

Borovsky et al. selected the peppers with violet and green immature fruits as the parents to develop the F_2_ population. *CaMYB* co-segregated with *A* was identified, which is characteristic of the R2R3-type MYB gene family and closely related to pepper anthocyanin synthesis [19]. The expression analysis of *A* in plant tissues showed that despite the genomic sequence of *A* did not differ between violet and green fruits, *A* was specifically expressed only in violet fruits. It was hypothesized that the absence expression of *A* in green fruits was due to the variation in the promoter region. Zhang et al. acquired the faded pepper fruit with poor anthocyanin accumulation by silencing *CaMYB* in purple peppers, which verified the role of *CaMYB* in anthocyanin metabolism in peppers [72]. They found that the accumulation of anthocyanin was high in the Z1-1-Z1-3 stage (18–30 days after flowering) of pepper fruit development, and was positively correlated with the expression of *CaMYB*. In addition, it was shown that the expression level of *CaMYB* was up-regulated in peppers under low-temperature stress. In contrast, the accumulation of anthocyanin pigments in pepper fruits was significantly higher than normal [83,84]. Researchers also obtained pepper fruits with significantly reduced anthocyanin content by silencing *CaWD40* in pepper, demonstrating that in addition to MYB, the WDR (WD40 repeat proteins) protein family also regulates anthocyanin biosynthesis in pepper [85].

## 4. Research of the Mature Fruit Color in Pepper

The color variation of ripe pepper fruits is abundant. Researchers have continued investigating the genes that control the colors of ripe pepper fruits, laying the foundation for breeding better quality pepper varieties and clarifying the specific molecular regulatory mechanisms of pepper fruit color (Table 2).

### 4.1. Research of Red, Yellow, and Orange Mature Fruit Related Genes in Pepper

The color conversion period of pepper fruit from immature to mature fruit is related to the degradation of chlorophyll and carotenoid synthesis in pepper fruits. The genes involved in carotenoid biosynthesis were identified (Table 3). Guo et al. performed the qRT-PCR analysis of 12 different pepper materials and found that *GGPS*, *PSY*, *CRTZ-2*, and *CCS* are essential in the biosynthesis of carotenoids in mature pepper fruits [86]. The expression levels of these genes were correlated with each other. Early genetic studies on peppers suggested that the mature fruit color of peppers was controlled by a single gene *Y*. The red allele is dominant to the yellow allele, and the genotype of red fruit is *Y*, while the genotype of yellow fruit is *y* [87]. Hernandez et al. established an F_2_ segregated population of peppers with red and white ripe fruits in 1985. Eight expected pepper fruit color phenotypes were observed in the F_2_ population, proving that the color of pepper ripe fruit was controlled by three pairs of mutually independent loci: *c1*, *c2*, and *y* [88]. Different combinations of the three loci constitute different colors of pepper ripe fruit, from red to white. It was proposed that pepper ripe fruit exhibited white when all three loci were recessive and red when all three loci were dominant [88]. Subsequently, Smith and Thorup also confirmed that the ripe fruit color of pepper is controlled by three pairs of genes: *c1*, *c2*, and *y* [38,89]. They also claimed that *c1* has a significant effect on carotenoid content, but the results of this study did not precisely match Hernandez’s genetic model of fruit color.

Houlné and Deruere isolated the cDNA and gDNA of a gene that is specifically highly expressed in the later-maturing pepper fruits [90,91]. The protein encoded by this gene was an oxidoreductase, which was involved in the synthesis of capsaicin and was present only in the mature red pepper fruit but not the yellow fruit. Bouvier et al. initially isolated *CCS* and obtained its cDNA sequence (1750 bp), which catalyzes the conversion of antheraxanthin to capsanthin and capsorubin from pepper [92]. Based on phenotype data from F_1_ and F_2_ populations, Lefebvre et al. concluded that the red phenotype was dominant over the yellow phenotype [93]. *CCS* was co-segregated with the red fruit trait, suggesting that *CCS* and *Y* might be the same gene controlling the red pepper fruit formation. Further analysis showed that yellow fruit might be related to the deletion of *CCS*. This analysis agreed with the findings of Bouvier et al., who discovered *CCS* synthase only in red maturity peppers but not in yellow fruits [19]. Popovsky et al. mapped *CCS* on chromosome 6 in pepper with different hybrid populations. *CCS* was detected only in red fruits but not in yellow fruits. The research verified that *CCS* was co-segregated with the *y* locus, so it was determined that *CCS* and *y* were the same genes [94].

Although earlier researchers generally attributed yellow-ripening pepper fruits to the deletion of *CCS*, an increasing number of studies have shown that the genes regulating yellow-ripening pepper fruits are not limited to the deletion of the *CCS* gene, and different pepper materials resulted in different fruit color variations. Popovsk et al. found that the yellow color of the ripe pepper was due to a 240 bp base deletion at the 5′ end of the *CCS* gene [94]. Ha et al. found *CCS* expression in the yellow pepper materials without haematochrome accumulation. After the sequence alignment of the *CCS* coding region, they found that the reason for the yellow color of the materials was a single base mutation in *CCS* [95]. It caused early expression of termination codons or a code-shifting mutation that prematurely terminated the synthesis of capsanthin. Both Li and Tian identified *CCS* in the yellow pepper fruit material CK7. They discovered that the fruit showed a yellow color due to the mutation of the *CCS* start codon that advanced the stop codon and the base deletion of *CCS*. In summary, it was demonstrated that *CCS* variation was different in different yellow fruit materials [96,97].

Hernandez and Thorup suggested that orange ripe pepper fruit was regulated by the *c2* locus [88,89]. Huh et al. established an F_2_ population with the cross of TF68 (Red Fruit) and Habanero (Orange Fruit) to identify genes and marker loci closely linked to the red/orange pepper fruit phenotype. They selected eight genes related to the carotenoid biosynthesis process of pepper: *PSY*, *FPS*, *GGPS*, *PDS*, *LCY*, and *CCS*, and found that only *PSY* was completely co-segregated with fruit color in the F_2_ population. Further analysis revealed that *PSY* was the gene responsible for fruit color development and was not linked with other candidate genes. Therefore, they hypothesized that *PSY* might be a candidate gene at the *c2* locus and that it was located on chromosome 7 in pepper. Kim et al. found that alternative splicing of *PSY* in intron 5 of orange ripening pepper fruits leads to early termination of transcription, thus further demonstrating the co-segregation of *PSY* with the *c2* locus [98]. Tian et al. obtained orange fruits by silencing *PSY* in pepper, further verifying the function of *PSY* [97]. Moreover, other research detected *PSY* in both red and orange pepper fruits, so it was inferred that *PSY* was not the main cause of the color variation between red and orange pepper fruits [79]. At the same time, Borovsky et al. obtained an orange mutant by EMS mutagenesis, which was caused by a single base mutation (A/G) at 709 bp in *CHY2*, so they inferred that *PSY* was not the only gene responsible for the difference between red and orange fruits, and that orange pepper fruits might also be regulated by *CHY2* [99]. A study has reported that the *c1* locus may affect carotenoid content rather than type in pepper fruits at maturity. In turn, the *c1* locus may also affect the reduction of pigmentation in red, orange, and yellow fruits. Unfortunately, the specific regulatory mechanism regarding this point has not been well explained [100].

### 4.2. Research of White, Green, and Brown Mature Fruit Related Genes in Pepper

Additionally, Some specific fruit color genes in pepper were also identified (Table 4). Lee et al. selected orange pepper ‘Habanero Orange’ (HO) and white pepper ‘Habanero Peach’ (HP) as parental lines to develop a genetic group. The contents of chlorophyll, carotenoid, and total soluble solids in HP genotype mature fruits were reduced, and there was no mutation of *y* in HP and HO, but there was a mutation at the *c2* locus [100]. In the F_2_ population, an SNP marker on chromosome 1 was significantly associated with the white fruit phenotype using BSA-seq. Further analysis revealed that *PRR2* was near this SNP marker and co-segregated with the white fruit phenotype. In conclusion, *PRR2* is a candidate gene for the *c1* locus.

The olive green and brown coloration of pepper fruits at maturity is on account of the simultaneous presence of green, red, or yellow pigments in fruits. The superposition of pigments makes the mature fruits appear brown and olive green [21,27]. In 1950, Smith et al. obtained a brown fruit from the hybrid combination of green and red pepper. Mature pepper fruits with green and brown colors were acquired by crossbreeding with another yellow pepper. Smith found that *cl* can hinder chlorophyll degradation through progeny phenotype analysis [38]. Borovsky et al. cloned a stay-green gene, *CaSGR*, in pepper. A single nucleotide mutation in the gene sequence blocked the chlorophyll degradation process during the color change from immature to mature. Genetic analysis indicated that *CaSGR* and *cl* co-segregated and concluded that the *CaSGR* gene is the *cl* gene [61]. Barry et al. also confirmed that *CaSGR* and *cl* are the same genes [101]. Since the previous research on *CaSGR* has been limited, its regulatory mechanisms remain unknown.

*CarbcL*, located on chromosome 12 in pepper, is another gene affecting chlorophyll and capsaicin synthesis, except for the abovementioned genes. *CarbcL* is a light-sensitive gene whose expression significantly increases when light stimulates [102]. However, there are fewer studies on *CaSGR* and *CarbcL*, and their specific regulatory mechanisms during pepper fruit development are still unclear and need to be continued.

In conclusion, the inheritance of pepper fruit color is a quantitative trait inheritance with multiple genes acting together. Although some progress has been made in the research of pepper fruit color, the clear genetic mechanism and regulatory mechanism have not been effectively elucidated.

## 5. Conclusions

Fruit color is one of the most direct economic characteristics of pepper. It is also an important factor in determining consumer acceptance and breeder preference. Researchers have been studying the fruit color of peppers since the 1930s. More and more reports have revealed that colorful pepper fruits are rich in various pigments, such as chlorophyll, carotenoids, and anthocyanins. Scholars also established the complex biosynthetic pathway process system of each pigment. They used it as a basis to research some important metabolic enzymes and coding genes in the pathway. However, pepper color is co-regulated by a variety of pigments, which is a complex regulatory system. More abundant regulatory networks need to be explored by advanced biotechnology and genetic research methods. In addition, pigment anabolism is affected by various environmental conditions, but the regulatory mechanism of environmental factors is still unclear. Moreover, pepper fruits contain a variety of pigments, among which there are still many unknown pigments that need to be further understood.

In recent years, with the help of the development of high-throughput molecular biotechnology and the selection of pepper resources with specific color traits, many scholars have already conducted identification and functional analysis of genes that play a crucial regulatory role in the formation of pepper fruit color. A preliminary understanding of the molecular genetic mechanism of pepper fruit color was obtained. In pepper fruits, *LOL1*, *GLK2*, *APRR2*, and *SGR* genes are related to chlorophyll synthesis and degradation; *PSY* and *CCS* are involved in carotenoid synthesis; *CaMYB* is related to anthocyanin synthesis. However, the number of genes related to pepper fruit color that have been detectd is limited. And there are relatively few reports on the specific regulatory mechanisms of related genes. More structural genes and regulatory genes involved in the synthesis and metabolism of pepper fruit pigment also need to be discovered.

Most of the peppers sold on the market are immature fruits, immature fruit color is an essential trait that determines the appearance and commercial quality. But now a majority of studies on pepper fruit color have focused on ripe fruit, and relatively few studies have been conducted on immature pepper fruits. Peppers with rare fruit colors can provide high-quality germplasm resources for breeders and increase the genetic diversity of peppers. Therefore, future research on immature and rare pepper fruit colors should be strengthened.

Future research on pepper fruit color should focus on the following aspects: (1) With the rapid development of high-throughput omics technology in recent years, the omics research on the biosynthesis of pepper fruit pigments should be strengthened to find and isolate other unknown pigments in pepper fruits, so as to provide theoretical basis for further detailed description of pepper fruit color formation. (2) It is necessary to strengthen the research on the transcriptional regulation mechanism of pepper pigment biosynthesis, which will help us to understand the transcriptional regulation mechanism of pepper pigment biosynthesis more deeply. (3) The collection and evaluation of germplasm resources with different fruit colors should be strengthened, and the linked molecular markers should be developed to lay the foundation for the study of genetic mechanism of pepper fruit color and the selection of new varieties.

## Figures and Tables

**Table 1:** Enzymes and genes involved in chlorophyll biosynthesis of pepper.

Short Name	Enzyme Name	Chromosome Location	Gene ID
GluTR	Glutamyl-tRNA reductase	4	LOC107867325
		8	LOC107840125
GSA-AM	Glutamate-1-semialdehyde 2,1-aminomutase	5	LOC107863035
PBGS (ALAD)	Porphobilinogen synthase (5-Aminolevulinate dehydratase)	1	LOC107856090
PBGD	Porphobilinogen deaminase (Hydroxymethylbilane synthase)	7	LOC107878895
UROS	Uroporphyrinogen III synthase (Uroporphyrinogen III co-synthase)	4	LOC107867127
UROD	Uroporphyrinogen III decarboxylase	6	LOC107875768
		10	LOC107845519
CPOX	Coproporphyrinogen III oxidase	10	LOC107845388
PPOX	Protoporphyrinogen oxidase	1	LOC107840071
MgCh	Magnesium chelatase H subunit	5	LOC107870073
	Magnesium chelatase I subunit	10	LOC107845383
	Magnesium chelatase D subunit	5	LOC107870054
MgPMT	Magnesium proto IX methyltransferase	3	LOC107862118
MgPEC	Mg-protoporphyrin monomethylester	10	LOC107844048
DVR	3,8-Divinyl protochlorophyllide a 8-vinyl reductase	1	LOC107842746
POR	Protochlorophyllide oxidoreductase	3	LOC107863251
CHLG	Chlorophyll synthase	9	LOC107842693
CAO	Chlorophyllide a oxygenase	6	LOC107872996

**Table 2:** Enzymes and genes involved in anthocyanin biosynthesis of pepper.

Short Name	Enzyme Name	Gene Name	Chromosome Location	Gene ID
PAL	Phenylalanine ammonia-lyase	PAL1	9	LOC107843092
C4H	Cinnamate 4-hydroxy-lase	C4H	6	LOC107875406
4CL	4-counmaric CoA ligase	4CL	3	LOC107862076
CHS	Chalcone synthase	CHS	5	LOC107871256
CHI	Chalcone isomeras	CHI	11	LOC107852750
F3H	Flavanone-3-hydroxylase	F3H	2	LOC107859880
DFR	Dihydroflavonol-4-reductase		2	LOC107860031
ANS	Anthocyanidin synthase	OXR1	10	LOC107843451
UFGT	Flavonoid3-O-glucosyltransferase	UFGT	10	LOC107843659
MT	Methyltransferase	PMT2	1	LOC107877679
RT	Rhamnosyltransferase		3	LOC107862351
AT	Acyltransferase		3	LOC107863748
GST	Glutathione S-transferase	GST	9	LOC107842862

**Table 3:** Enzymes and genes involved in carotenoid biosynthesis of pepper.

Short Name	Enzyme Name	Gene Name	Chromosome Location	Gene ID
GGPS	Geranylgeranyl pyrophosphosphate synthase	*Ggps*	4	LOC107867046
PSY	Phytoene synthase	*Psy*	4	LOC107868281
PDS	Phytoene desaturase	*Pds*	3	LOC107861625
ZDS	ζ-carotene desaturase	*Zds*	8	LOC107839468
LYC	Lycopene cyclase	*CrtL*	5	LOC107869983
CRTZ	β-carotene hydroxylase	*CrtZ-2*	3	LOC107863219
		*CrtZ-1*	6	LOC107873401
Ze	Zeaxanthin epoxidase	*Ze*	2	LOC107860302
CCS	Capsanthin-capsorubin synthase	*Ccs*	6	LOC107875664

**Table 4:** Some identified fruit color genes in pepper.

Gene Name	Color	Functional Analysis	Chromosome
*pc1* (*pc8.1*, *LOL1*)	Green, white	Affect chlorophyll content	1
*pc10.1* (*CaGLK2*)	Green, white	Involved in chloroplast development	2
*APRR2-Like*	Green, white	Affect chlorophyll content	2
*A* (*CaMYB*)	Violet, nearly black	Regulate anthocyanin synthesis	10
*MoA*	Violet	Adjust the shade of violet	11
*CCS* (*Y^+^*)	Red, yellow	Regulate capsaicin synthesis	6
*PSY* (*C2*)	Orange	Regulate carotenoid synthesis	7
*CaPRR2* (*C1*)	White, green, brown	Regulate carotenoid synthesis	1
*CaSGR*	White, green, brown	Chlorophyll degradation	1
*CarbcL*	White, green, brown	Regulate chlorophyll and capsaicin synthesis	12

## Data Availability

Not applicable.
